# Against the Odds: Management of Ureteral Calculus in Patient With Crossed Fused Renal Ectopia

**DOI:** 10.7759/cureus.10895

**Published:** 2020-10-11

**Authors:** Jasmin Alić, Zahid Lepara, Hajrudin Spahović, Senad Bajramović, Jasmina Heljić

**Affiliations:** 1 Urology Clinic, Clinical Center University of Sarajevo, Sarajevo, BIH; 2 Pediatrics, General Hospital "Prim. Dr. Abdulah Nakaš", Sarajevo, BIH

**Keywords:** congenital anomalies of kidney, fused kidney, crossed fused renal ectopia, ureteral stone, eswl (extracorporeal shockwave lithotripsy)

## Abstract

Crossed fused renal ectopia (CFRE) is a rare congenital abnormality of the urinary tract where the kidneys are fused on one side, while the ureter of the ectopic kidney crosses the midline with the normal entrance in the bladder on the contralateral side. Congenital anomalies are associated with a stone formation whose management represents a real challenge. To our knowledge, we report the second case of CFRE associated with ureteral stone, which has been successfully resolved with Extracorporeal Shockwave Lithotripsy (ESWL) and the first of its kind where a sufficient degree of stone disintegration has been achieved after a single session with a complete stone clearance during the follow-up. Radiological examination showed an inferior type of CFRE with stone in the proximal part of the ureter of the upper kidney. ESWL is an acceptable and effective treatment option in CFRE patients due to the minimally invasive approach, potentially high stone-free rate, and rare complications.

## Introduction

Crossed fused renal ectopia (CFRE) is a rare congenital abnormality of the urinary tract where one of the kidneys is located on the opposite side of its vesicoureteral junction, fused to the ipsilateral kidney. The incidence has been calculated at one in 2000 cases with a slight male predominance [1]. Most individuals have no symptoms in absence of infection and/or urolithiasis, thus CFRE is often incidentally detected. It is associated with an increased rate of stone formation, but also with skeletal, cardiovascular, genitourinary, and gastrointestinal abnormalities [2]. A case of ureteral calculus in a patient with CFRE successfully treated with only one session of Extracorporeal Shockwave Lithotripsy (ESWL) is presented in this report.

## Case presentation

**Figure 1:**
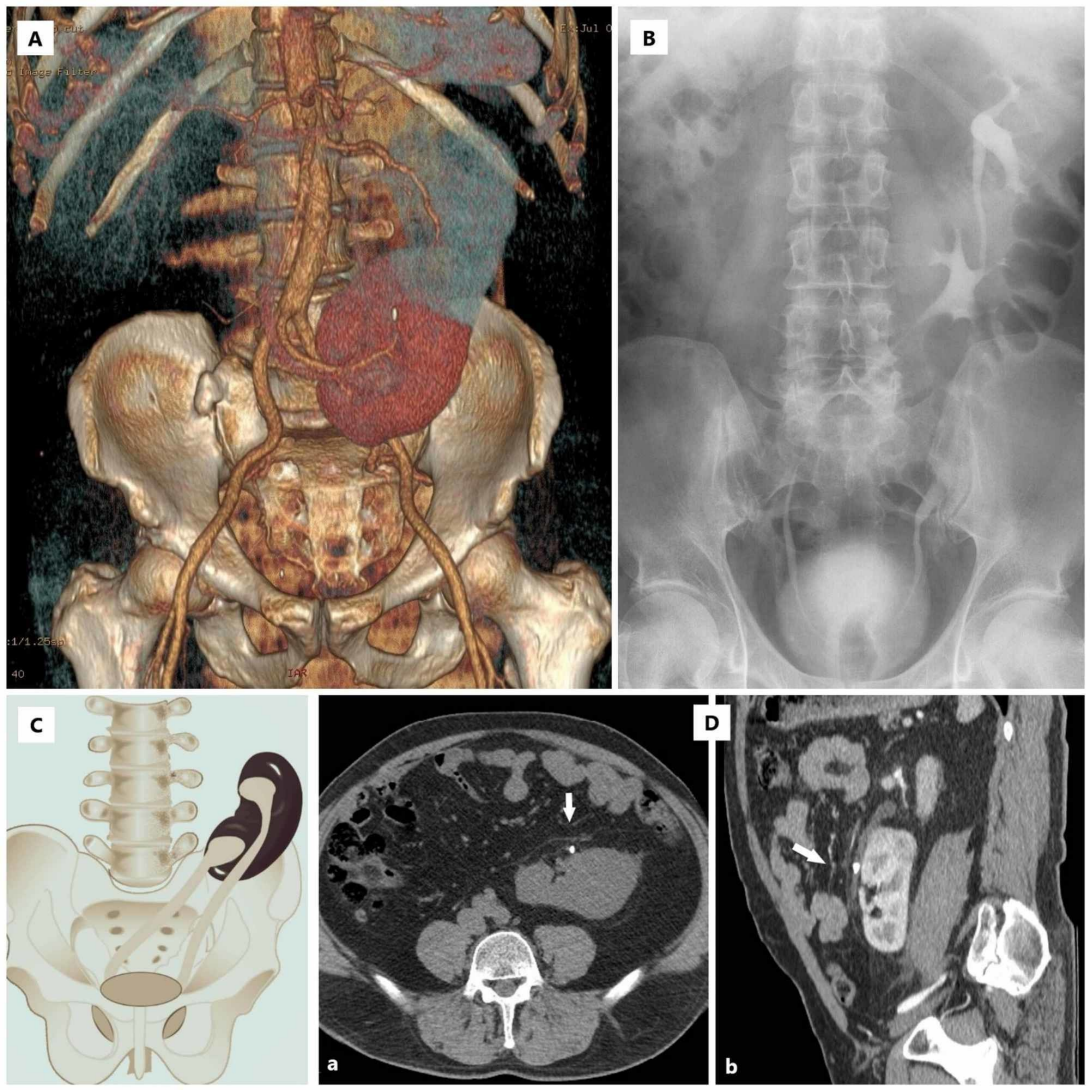
Radiological features of present case (A) 3D computed tomography revealed inferior right-to-left crossed fused renal ectopia with the abnormal arterial supply and stone in the projection of proximal ureter of the upper kidney; (B) Postoperative IVU showed no stone shadows or signs of obstruction. The ureter of ectopic kidney crosses the midline and enters into the bladder at the normal position; (C) Abnormal anatomy of the urinary tract in the present case (scheme); (D) Preoperative abdominal CT scans in axial (a) and sagittal (b) plane revealed stone (arrow) in the proximal part of the ureter of the upper kidney. IVU = intravenous urography, CT = computed tomography.

A 53-year-old man presented with intermittent abdominal pain projecting into the left lumbar region. His blood pressure has been successfully regulated with antihypertensive drugs (lisinopril plus amlodipine). Physical examination revealed left lumbar tenderness, without any other clinically significant observations. The renal function, hemogram, and urine analysis showed no abnormalities. Initial abdominal ultrasonography revealed an absent kidney in the right lumbar lodge and a solitary left kidney with two collecting ducts. The examination was supplemented with the plain of kidney-ureter-bladder (KUB) and contrast-enhanced computed tomography (CECT). CECT scan showed inferior CFRE with stone at 3 centimeters distal to the ureteropelvic junction of the upper (left) kidney (Figure 1).

The crossed ectopic kidney was located inferior to the left kidney with renal sinus oriented craniomedially. Stone size was 9.2 x 5.9 mm, and mild dilatation of the upper renal collecting system was observed, with slower contrast agent secretion. The average stone density measured by CT was 1090 Hounsfield units (HU). Both upper and lower kidney had one renal artery originating from the unusual place on the aorta at the level of the superior mesenteric artery and aortic bifurcation (Figure 1A). Renal Color Doppler ultrasonography excluded the renovascular etiology of hypertension. Renal resistive index values in both renal arteries were in the normal range.

After being presented with the benefits and risks, the patient agreed to undergo ESWL treatment. Previously, medical expulsive therapy (tamsulosin, cps. 0.4 mg, once per day) and conservative treatment for pain relief were prescribed.

A single ESWL session was performed using the Dornier Compact Delta II unit (Dornier Medizintechnik GmbH, München, Germany). The generator voltage was 19 kV and a total of 2500 shockwaves at a delivery rate of 90 pulses were delivered without any form of anesthesia. During the treatment, the stone was visualized by fluoroscopy and ultrasonography. A sufficient degree of stone disintegration has been achieved and the patient was discharged after three days. Stone composition analysis could not be performed due to the inability to collect small fragments.

To confirm complete stone clearance, we performed intravenous urography (IVU) which showed no residual fragments, with the adequate secretion of contrast agent in both kidneys (Figure 1B). The patient stayed asymptomatic with no dilatation of the renal collecting systems detected by abdominal ultrasonography.

## Discussion

Crossed renal ectopia is one of the rarest urinary tract anomalies, occurring once in every 7500 autopsies or 3078 CT scans [2,3]. The precise mechanism of renal fusion anomalies development is not fully clarified, but ureteral and mechanical theories have been proposed [4].

Renal ectopic anomalies are classified into four types: crossed ectopia with and without fusion, solitary, and bilaterally crossed ectopia [5]. Subsequently, 6 subtypes of CFRE are described: inferior ectopia, sigmoid or S-shaped kidney, lump kidney, L-shaped or tandem kidney, disc kidney, and superior ectopia [1,5]. The most common type of renal fusion is inferior ectopia with the upper pole of the ectopic kidney fusing with the lower pole of the normal kidney [6]. It seems that left-to-right crossover occurs more frequently [2,6]. Our report describes the uncommon finding of crossed right-to-left inferior renal ectopia with both kidneys placed on the left side of the body associated with proximal ureteral calculus.

Most patients are incidentally diagnosed during routine radiological and surgical procedures. When symptoms occur, they include abdominal or flank pain, hematuria, and urinary tract infection. It is notable that the pain caused by the calculi in a crossed ureter is manifested on the side of the embryonic origin. However, the pain resulting from renal parenchyma involvement is reflected on the side of the ectopic kidney [1].

CFRE can be associated with other anomalies, urological conditions, or malignancies. Solanki et al. reported urological anomalies in all 6 patients in their series [7]. In children, CFRE is often found during diagnostic evaluation of syndromes and is often associated with other urinary tract anomalies, such as pelvi-ureteric junction obstruction (PUJO), ureteric strictures, vesicoureteral reflux (VUR), megaureter, and renal dysplasia. Furthermore, this congenital anomaly sometimes appears combined with non-urinary tract anomalies. Loganathan and Bal, in their study of 36 pediatric patients, found anorectal anomalies to be predominantly associated with CFRE [8]. In our case, a radiological examination did not reveal any associated urological anomaly.

Management of urolithiasis in patients with CFRE is technically difficult due to the abnormal position, vascular supply, and relations with surrounding structures. Subsequently, the usual treatment may not be as efficient [2]. Up to today, there is no standardized guideline for the management of upper urinary tract stones in CFRE [6]. Few studies have reported the treatment of kidney and ureteral calculi with ESWL, percutaneous nephrolithotomy (PCNL), laparoscopic nephrolithotomy (LNL), and retrograde intrarenal surgery (RIRS). An open surgical approach is avoided due to the variability of CFRE forms and associated blood vessel anomalies [2,9]. ESWL is otherwise considered safe and effective in the treatment of stones in almost all parts of the urinary tract, but when it comes to congenital anomalies, the results are contradictory.

After reviewing the available literature, a total of 35 CFRE cases associated with stones were found. However, only five patients with ureteral stones were reported. Only seven patients were treated with ESWL, with three reported failures where an alternative method was required [9].

To the best of our knowledge, only one case of CFRE involving ureteral calculi treated with ESWL has been reported to date. Kato et al. performed the first successful ESWL of ureteral stone in a patient diagnosed as S-shaped CFRE and calculus in the proximal ureter of the crossed kidney. Stone-free status has been achieved after two sessions with previous ureteral stent insertion [10]. Kodama et al. performed successful ESWL treatment of proximal ureteral stone in crossed, but unfused renal ectopia with complete stone clearance after one session [11]. Another study reported 2 patients with an L-shaped CFRE treated with ESWL for renal stones. The first patient was stone-free after three sessions, while the second patient treatment failed [12]. 

Residual fragments in the form of steinstrasse formation after the ESWL of renal stones were reported [13]. Additional three cases with a history of failed ESWL treatment of renal stones have been identified [2]. Complete stone clearance was achieved in 14 patients, including all treatment modalities (ESWL, PCNL, LNL, RIRS). They concluded that conservative treatment and ESWL of upper urinary tract calculi in patients with CFRE are insufficient. They advised ureteroscopy as the first choice and emphasized that RIRS may become the first-line treatment for renal and unreachable ureteral stones [2].

Tunc et al. performed ESWL treatment of 150 patients with anomalous kidney, of which there were only four CFRE cases. The overall stone-free rate was 57%, but only one patient achieved a stone-free status after three months. Authors suggest that the anomaly type, stone composition, and localization remain the main parameters affecting the treatment success [14].

Hounsfield units have been used to predict the stone composition and the success of ESWL treatment [15]. In our case, density analysis suggests the calcium oxalate composition, with low disintegration probability. However, a single ESWL session was sufficient to achieve no detectable stone fragments.

Despite difficulties in positioning and identifying the stone, there was no need for ureteral stent placement. ESWL was performed as an urgent procedure after excluding the ureteral anatomic obstruction. The achieved result seems even more surprising considering its position and flow across the anterior surface of the lower kidney and its hilum, and over the aberrant blood vessels.

## Conclusions

ESWL could be a primary option for the ureterolithiasis in patients with CFRE, but each treatment decision should be individual due to technical difficulties. ESWL is an acceptable and effective treatment option in these patients due to the minimally invasive approach, potentially high stone-free rate, and rare complications.
